# Imperfection of the Heart—Discoloration of the Skin—Post-Mortem Appearances

**Published:** 1829-07-01

**Authors:** 


### VIII.

### Imperfection of the Heart—Discoloration of the Skin—Disorder of the Stomach—Death—Dissection.

In the 13th volume of the Medico-Chirurgical Transactions, page 215—and in the 6th volume of this Journal, (April 1827,) page 343, will be found a curious case of disease of the heart, accompanied by a partial discoloration of the skin, by Dr. J. Johnson. A case somewhat analogous has recently occurred in Dr. Johnson's practice. In the first case, (the lady of an eminent barrister) the right ventricle of the heart was exceedingly *extenuated in its parietes*, which were not more than the seventh of an inch in thickness. The *capacity* of the left ventricle, on the other hand, was so diminished, that it would not contain more than a couple of drachms of blood—its parietes being three quarters of an inch in thickness. The colour of the skin, in all parts exposed to the air, was as brown as that of a Mulatto, though the lady was very fair naturally. The pulse was so feeble, for many months previous to death, that it was scarcely perceptible at the wrist. The lady died exhausted, her great complaint being what she denominated "fits of heaving," quite different from vomiting. Her appetite was voracious—she had a sense of sinking at the pit of the stomach—and she was easily put out of breath on using any exertion. There was no other disease than that above described in the heart.

About four months ago a lady presented herself to Dr. Johnson, the colour of whose skin bore such a striking resemblance to that of the late Mrs. A. that his attention was instantly arrested by the similitude. His curiosity was still more excited when, on feeling the pulse, it had the same feeble silky beat as in Mrs. A.'s case. On further examination, there was found a considerable parallelism in other phenomena. Nausea and distress at the stomach were complained of, as the principal grievance, and dyspnœa was occasioned by the slightest exertion. The young lady could scarcely walk the length of a street, without exhaustion and breathlessness. The catamenia were irregular—the secretions depraved—the appetite capricious—the tongue loaded. It appeared that moral impressions of a sombre cast had been operating for two or three years on this lady's health, and the present symptoms had been gradually coming on for two years. On investigation, the dark colour of the skin was not entirely confined, as in Mrs. A's. case, to those parts of the body that were exposed to the air. The whole surface was darker than natural; but by no means of so deep a hue as the skin of the face, neck, and hands.

After some saline effervescing medicines had been given to tranquillize the stomach, and afterwards some alterative aperients to regulate the bowels, this lady took small doses of the sulphate of quinine, sometimes with soda—sometimes with infusion of roses and sulphuric acid. By these means her health improved greatly—the colour of the skin became less intense—the appetite increased—and she was able to walk two or three miles without fatigue. While thus improving, Miss C. went to a ball at Enfield, and danced. She was much worse afterwards, and came to town to consult Dr. J. He strenuously cautioned the young lady against a similar exertion in future; but, in despite of this advice, she went to another ball, two days afterwards, and danced six sets of quadrilles! She took to her bed immediately after this act of imprudence, and in less than three weeks she died. Mr. Hammond, of Lower Edmonton, attended the lady during this last illness, and Dr. J. saw her four or five times at her residence in the country. There had been some slight symptoms of pulmonary inflammation after the ball, which readily subsided; but gastric irritability, constant sense of sinking—languor—lassitude—faintness, (amounting almost to syncope) continued till within two or three days of her death, when coma supervened. Dr. J. at various times, examined the chest by means of the stethoscope—and once very particularly in the presence of Mr. Hammond. The respiration was clear and distinct every where. The action of the heart was also perfectly audible and regular; but there was no impulsion. The pulse at the wrist, during the time she had been improving under the tonic plan, had become much more developed; but during the last illness it continued very weak, and the colour of the skin became darker than ever.

Connecting the case with that of Mrs. A. above-mentioned, Dr. J. expressed his opinion to Mr. Hammond, that the heart was the organ most in fault. Dr. J. could not attend at the dissection; but his friend Dr. Hodgkin examined the body with his usual accuracy and ability, and favoured Dr. J. with copious notes of the dissection. Only a few extracts from these notes will be necessary in this place.

"Externally" says Dr. H. "the body was remarkable for its dusky hue. It was not merely the face which was so affected, but the neck, and particularly the hands, were in the same state. It appeared to depend on the deposit of colouring matter in the rete mucosum, and bore a close resemblance to the skin of a person in whom there is a slight admixture of the Ethiopean race."

There was nothing particular in the brain or its membranes, excepting that the convolutions were almost as much flattened as where there is a collection of fluid in the ventricles. Scarcely any serum was found in these cavities. The brain was rather softer than natural. The lungs were sound, and the pleura free from marks of inflammation.

"The pericardium was remarkably thin, transparent, and quite healthy. The heart was of *extremely small size*, and singularly *thin and flabby*. The *right* ventricle scarcely averaged the tenth of an inch in thickness—and the *left*, a quarter of an inch. The valves were healthy, except some partial translucent thickening of the mitral valve. There was a fibrinous coagulum, of very remarkable firmness, running for a considerable distance into the larger vessels." There was nothing else particular to be found, except that the right renal capsule was enlarged to about the size of the lobulus spigelii of the liver. It was readily seen on raising the concave surface of that viscus, and Dr. Hodgkin thinks it "not altogether impossible that it might, at times, have occasioned some pressure on the biliary ducts."

Now, although the pathological conditions of the heart, in Mrs. A.'s case, and in the one just detailed, are very different, yet the physiological disorders of function were nearly the same. The want of capacity in the ventricle connected with the general circulation—and of power in the parietes of the right ventricle, in Mrs. A. produced the same defective circulation throughout the pulmonary and aortic system, as the extremely small, thin, and flabby heart did in Miss C.'s case. It will hardly be doubted that, in both cases, the fatal event was dependent on the defects in the central organ of the circulation. But how, and whether the foregoing imperfections in the heart, were connected with the remarkable discoloration of the skin, we will not venture to say. The two cases, agreeing so strongly in the extreme weakness of the pulse, the dark hue of the skin—the affection of the stomach—and the physiological imperfection of the heart, may afford some information, at least as to prognosis, to others, when similar phenomena present themselves in practice. The remarkable amendment of health which took place in Miss C.'s case, under the use of tonics, is worthy of consideration. Had she not so imprudently exerted herself in the act of dancing, there is every reason to believe that life might have been preserved — at least for some years. When Mr. Hammond was called in, after the exertion above-mentioned, a great collapse and fainting fit occurred, from which the patient was, with difficulty, recovered. Is it not probable that the fibrinous concretion, or, what used to be called polypus, found in the heart and large vessels by Dr. Hodgkin, then took its origin? We think it very probable.

